# Right Ventricular Function in Patients With Significant Tricuspid Regurgitation

**DOI:** 10.31083/RCM47857

**Published:** 2026-02-26

**Authors:** Xavier Galloo, Philippe Unger

**Affiliations:** ^1^Department of Cardiology - Valve Clinic, Vrije Universiteit Brussel (VUB), Universitair Ziekenhuis Brussel (UZ Brussel), B-1090 Brussels, Belgium; ^2^Department of Cardiology, Université Libre de Bruxelles (ULB), CHU Saint-Pierre, B-1000 Brussels, Belgium

**Keywords:** right ventricular function, tricuspid valve, tricuspid valve insufficiency, ventricular remodeling, risk assessment, review

## Abstract

Significant tricuspid regurgitation (TR) is increasingly recognized as a major determinant of morbidity and mortality, yet the clinical impact of significant TR has long been underestimated. Assessment of right ventricular (RV) systolic function is central to understanding and managing TR and represents the principal determinant of symptoms, therapeutic response, and long-term outcomes. The unique sensitivity of the RV to alterations in preload and afterload leads to maladaptive remodeling, making accurate functional assessment essential for risk stratification and for optimizing the timing and type of intervention, especially given the expanding range of available surgical and transcatheter treatment options. Echocardiography remains the primary imaging modality, providing qualitative and quantitative evaluations of RV function through parameters such as tricuspid annular plane systolic excursion (TAPSE), RV fractional area change (RVFAC), and tissue Doppler systolic velocity (S′). Advances in speckle-tracking echocardiography for RV free-wall longitudinal strain and in three-dimensional imaging have improved accuracy; however, all echocardiographic measures remain limited by the complex geometry of the RV. When feasible and available, cardiac magnetic resonance (CMR) imaging serves as the reference standard for precise assessment of RV volumetric and functional parameters. Impaired RV systolic function, both before and after intervention, irrespective of the imaging parameter used for the assessment, consistently predicts adverse outcomes in patients with severe TR, including heart failure progression, reduced exercise tolerance, and decreased survival. Therefore, early recognition and quantification of RV dysfunction are crucial to enable timely therapy, as interventions before the development of advanced RV impairment provide symptomatic and survival benefits. This review summarizes the pathophysiology, quantitative thresholds, and prognostic significance of RV function assessment, emphasizing the pivotal role this evaluation plays in the contemporary management of significant TR.

## 1. Introduction

**Fig. 1.**
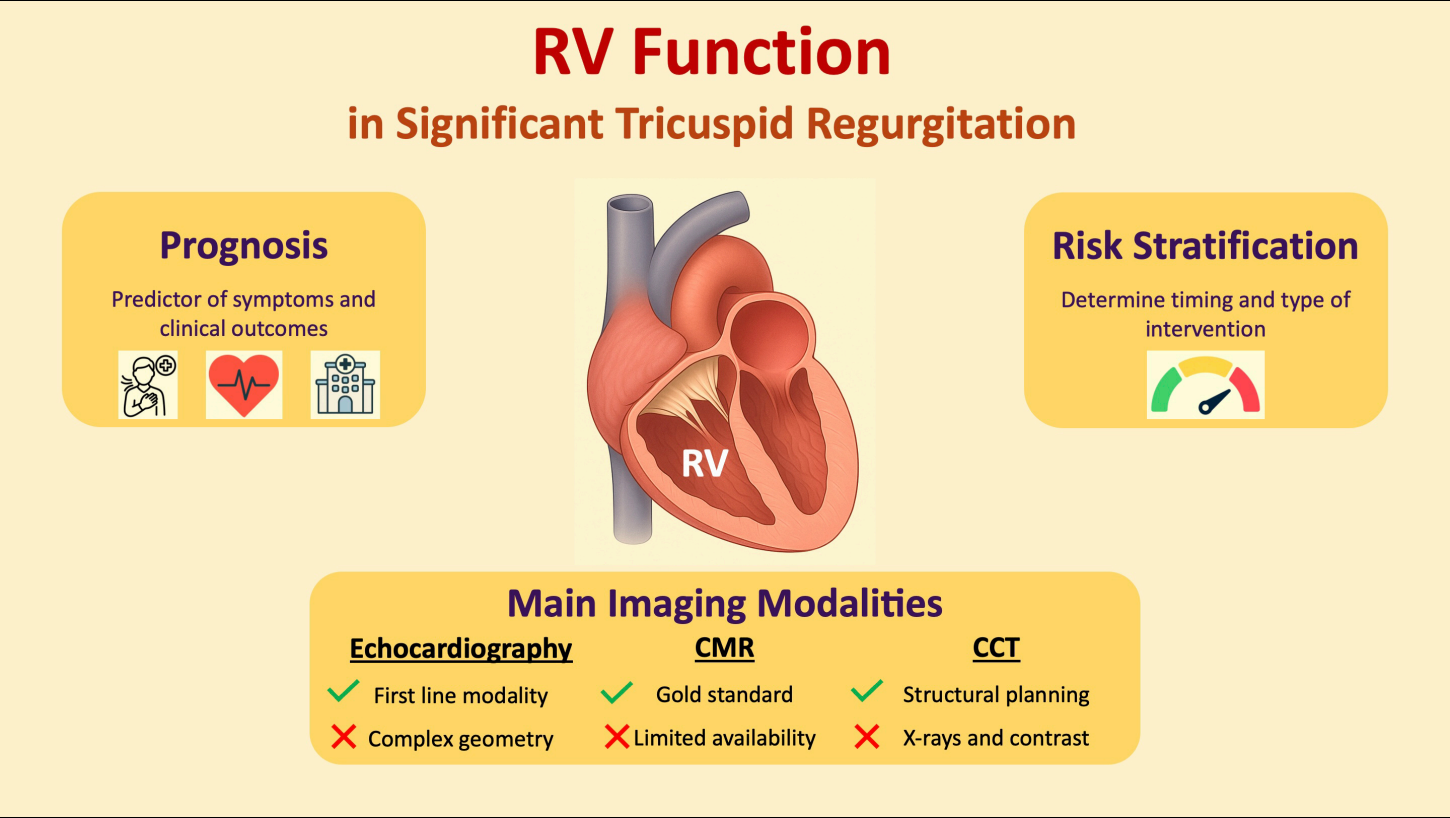
**Right ventricular function in patients with significant tricuspid regurgitation**. Abbreviations: CCT, cardiac computed tomography; CMR, cardiac magnetic resonance; RV, right ventricular.

For decades, clinical and research attention has largely focused on left ventricular (LV) structure and function, while the right ventricle (RV) was primarily considered as a conduit for pulmonary blood flow, rather than a contributor to systemic physiology. This concept slowed advances in our knowledge of the pathophysiology of right-sided heart disease [1]. Consequently, the tricuspid valve (TV) has long been regarded as the “forgotten heart valve” [2]. However, large population-based registries have demonstrated a 1.5–2% prevalence of significant (at least moderate) tricuspid regurgitation (TR) among the general population, with increasing prevalence of clinically-relevant TR with advancing age, reaching nearly 4% in individuals over 75 years of age [3, 4, 5]. There is increasing evidence from several patient cohorts that the presence of significant secondary TR (STR) has prognostic implications, and that, if left untreated, significant TR is associated with adverse clinical outcomes. These outcomes include quality of life, exercise capacity, mortality, and heart failure-related hospitalization, which are largely independent of LV and RV systolic function and of the presence of pulmonary hypertension (PHT) [5, 6, 7, 8]. Surgical and transcatheter TV interventions are increasingly available for the treatment of significant TR but are still underused. One of the major clinical challenges is to determine the optimal timing for intervention, because patients may remain asymptomatic for a long time if receiving adequate diuretic treatment, and referrals often occur when patients have advanced right heart failure and irreversible end-organ dysfunction [9, 10, 11]. Current guidelines for the management of valvular heart disease issued by the European Society of Cardiology (ESC) [12] and by the American Heart Association/American College of Cardiology (AHA/ACC) [13] recommend intervention based on a combination of clinical and echocardiographic factors, including TR severity, symptom burden, anatomical parameters such as tricuspid annular dilation, and, importantly, the presence and extent of PHT and RV dysfunction. Nonetheless, evaluating the right heart in the setting of TR remains challenging in current clinical practice, because of the complex three-dimensional anatomy resulting in difficult image acquisition, specific right-sided hemodynamic patterns, the load dependency of common RV indices, and the complex interplay between the RV, the pulmonary vasculature, and the LV [1]. This review provides an overview of current evidence on the assessment, clinical features, and prognostic impact of right heart function in patients with significant TR (Fig. 1).

## 2. The Normal Right Ventricle

The RV is located anteriorly within the thoracic cavity, directly behind the sternum. It is bordered by the TV annulus (TA) proximally and by the pulmonary valve distally. The RV is classically divided into three structural components: (1) the inlet region, which comprises the TV apparatus, including the leaflets, chordae tendineae, and papillary muscles; (2) the trabeculated apical myocardium; and (3) the infundibulum, also known as the conus arteriosus, i.e., the smooth-walled outflow tract that directs blood toward the pulmonary valve [1, 14]. For functional and imaging purposes, the RV can also be divided into anterior, lateral, and inferior walls, as well as into basal, midventricular, and apical segments [1].

The RV has a unique, complex, three-dimensional geometry. Whereas the LV is ellipsoid, the RV has a triangular shape in the sagittal plane and a crescent-shaped profile in cross-sectional views [1]. Under normal loading conditions and in the absence of conduction abnormalities, the interventricular septum is concave toward the LV during both systole and diastole. In adults, the RV end-diastolic volume typically exceeds that of the LV [1]. However, due to thinner myocardial walls, the RV’s mass is approximately one-sixth that of the LV [15].

The RV myocardium is composed of two main muscle layers: a superficial and a deep one. Superficial fibers run parallel to the atrioventricular (AV) groove and are oriented circumferentially [14, 16]. On the sternocostal surface, the superficial fibers adopt an oblique trajectory towards the apex and continue into the superficial myocardial layer of the LV, reflecting the myocardial fiber continuity between the ventricles. In contrast, the deep RV muscle fibers are arranged longitudinally, extending from the base to the apex; they are primarily responsible for the longitudinal shortening that characterizes normal RV contraction [1, 14, 16].

## 3. Pathophysiology and Clinical Presentation of RV Dysfunction in TR

Normal RV function is the result of the precise interaction of several factors, including systemic venous return (preload), intrinsic myocardial systolic and diastolic performance, pulmonary vascular resistance (afterload), and pericardial compliance. Under physiological conditions, RV systolic function is primarily driven by the shortening of longitudinal myocardial fibers, accounting for about 80% of RV stroke volume [17]. However, in certain clinical scenarios, such as early after cardiac surgery, a transient but significant reduction in longitudinal function is often observed [18]. In these cases, the recruitment of circumferential myocardial fibers compensates for the impaired longitudinal mechanics, enabling overall cardiac output to be preserved [18].

The chronic volume overload of the RV observed in significant TR results from the addition of regurgitant flow to systemic venous return. This increases RV preload and consequently also RV afterload. In the early stages of chronic TR, the RV undergoes homeometric adaptation in accordance with Anrep’s effect, enhancing myocardial contractility while maintaining chamber dimensions and a relatively low end-systolic volume. This compensatory mechanism effectively preserves RV stroke volume and ejection fraction over a prolonged period despite ongoing volume overload [19, 20, 21]. However, as the severity of TR worsens, RV systolic function begins to decline, particularly in the longitudinal plane. The radial and anteroposterior components of contraction initially remain preserved. With further disease progression, a heterometric adaptation, as described by Starling’s law, becomes predominant. This phase is characterized by increases in RV end-diastolic and end-systolic volumes in an attempt to preserve forward stroke volume, albeit at the expense of adverse RV remodeling and RV hypertrophy. Although RV wall thickness may remain within normal limits, total RV mass increases. Eventually, elevated RV filling pressures and diastolic interventricular septal displacement (flattening) can impair LV filling and function, a phenomenon known as ventricular interdependence [19, 20, 21].

In patients with chronic significant TR, the substantial volume that regurgitates into the right atrium (RA) results in elevated RA pressures, which are subsequently transmitted to the systemic venous circulation, resulting in systemic venous congestion. This congestion manifests clinically as hepatomegaly, ascites, peripheral edema, and edema of the gastrointestinal tract [22]. Persistent venous congestion can further impair RV function through several mechanisms, including pericardial constraint, interventricular septal shift due to elevated RV diastolic pressure, and reduced coronary perfusion pressure; the latter particularly affects the subendocardial layers of the RV myocardium, making these layers more vulnerable to ischemia. This process initiates a vicious cycle in which progressive RV dysfunction promotes venous congestion, and prolonged congestion further deteriorates RV function. If left untreated, this cycle ultimately results in advanced, refractory right heart failure [22].

**Fig. 2.**
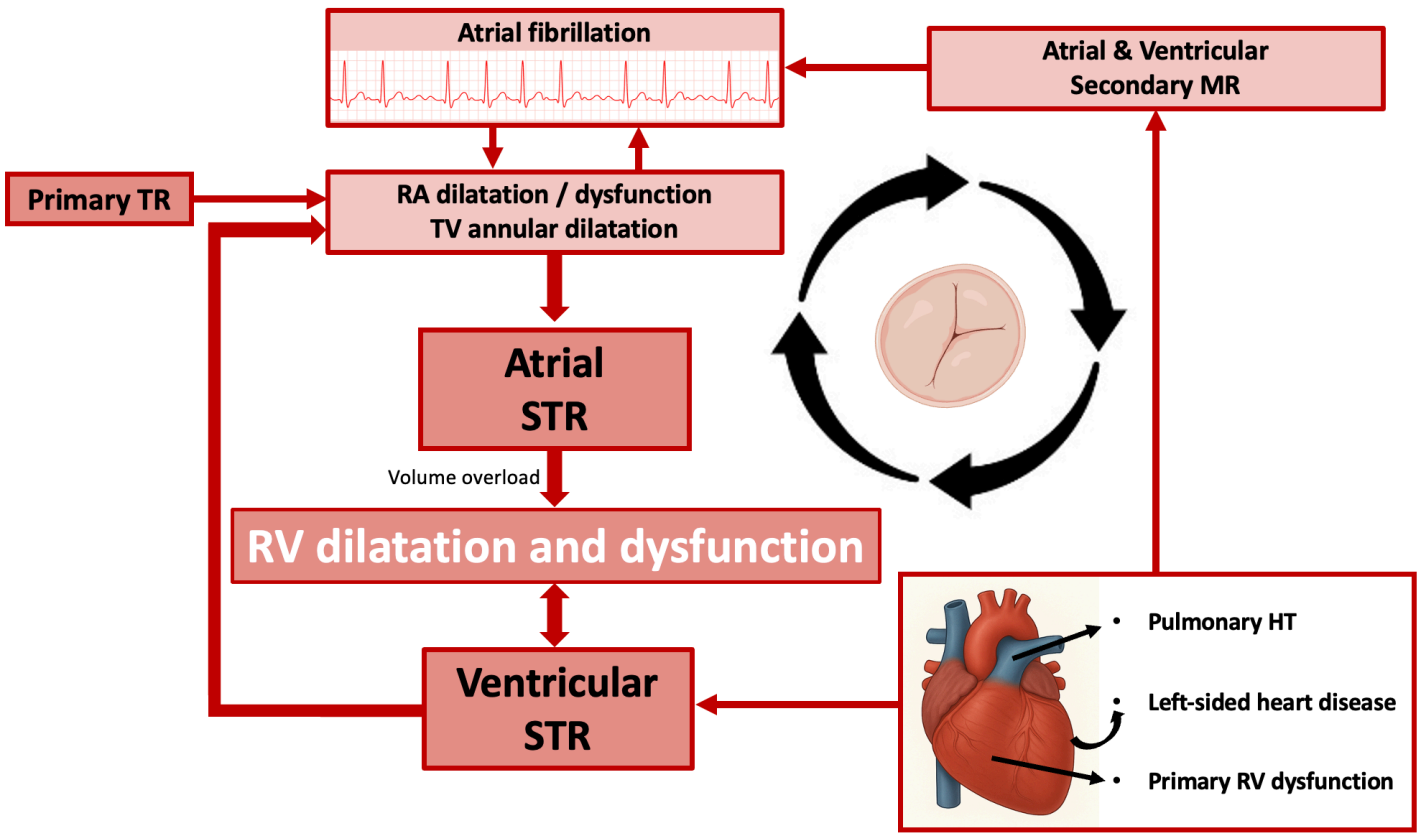
**Vicious circle of right ventricular dilatation and dysfunction in patients diagnosed with atrial and ventricular secondary tricuspid regurgitation**. Abbreviations: HT, hypertension; MR, mitral regurgitation; RA, right atrium; RV, right ventricle; STR, secondary tricuspid regurgitation; TR, tricuspid regurgitation; TV, tricuspid valve.

In 90% of cases, TR is the result of dilatation of the RA, RV, or TA, leading to STR. The most common form of STR is ventricular STR (VSTR), in which dilation of the RV causes tricuspid leaflet tethering during systole [23]. Atrial STR (ASTR), which occurs as a result of RA dilatation or dysfunction, often in the setting of atrial fibrillation, has long been neglected but has recently emerged as an important etiology of STR, accounting for 10%–15% of cases [24, 25]. There may be some overlap between VSTR and ASTR. Indeed, chronic significant VSTR may result in dilatation of the RA and TA due to volume overload (‘VSTR begets ASTR’), and chronic significant ASTR may eventually result in RV dilatation or dysfunction (‘ASTR begets VSTR’; Fig. 2) [23]. Therefore, in advanced disease, the chronic volume overload imposed by ASTR on the RV may have a deleterious effect on RV function, and some patients may present with complex forms of STR that have characteristics of both ASTR and VSTR [26].

Left-sided heart disease may also contribute substantially to the development of TR and subsequently to RV dysfunction (Fig. 2). In addition to left-sided valvular heart disease and to LV dysfunction, TR may also result from heart failure with preserved left ventricular ejection fraction (LVEF) (HFpEF), as a result of increased LV filling pressure, exercise-induced congestion, and, later, of pulmonary vascular disease. VSTR is a common phenotype in this setting. RV adaptation to the level of the pulmonary pressure (RV to pulmonary artery coupling (RVPAC)) is an independent prognostic factor in HFpEF [27]. However, atrial myopathy may also contribute to the development of STR [28]. Among patients with ASTR, the combination of AF and HFpEF is frequent and associated with poor outcomes [29].

The clinical presentation of RV dysfunction or failure is primarily related to systemic venous congestion and, in advanced cases, to low cardiac output. Venous congestion typically manifests as peripheral edema, jugular vein distention, hepatojugular reflux, ascites, painful hepatomegaly, and nocturia; in addition, as a result of congestion in the gastrointestinal tract, nausea and loss of appetite may occur. Low cardiac output induces fatigue, weakness, shortness of breath, chest pain/discomfort, dizziness and fainting. A S3 gallop may be heard upon auscultation. Weight gain as a result of fluid retention often indicates worsening heart failure and, in very advanced cases, jaundice and cachexia may occur. A prominent jugular V wave, pansystolic murmur at the lower left sternal border with inspiratory increase, and pulsatile liver are hallmarks of severe TR.

## 4. Principles and Challenges of RV Function Assessment

The assessment of RV function in patients with TR is particularly challenging as a result of the unique anatomy of the RV and its sensitivity to loading conditions. The main challenges result from the following: (1) The RV is a crescent-shaped structure with a broad base and a triangular apex, and includes outlet, apex, and inlet portions. When RV volume and pressure overload occur, as in significant TR, the RV loses its triangular shape and becomes more elliptical. Hence, geometrical assumptions are unreliable, making the assessment and interpretation of RV ejection fraction (RVEF) particularly challenging. Moreover, these anatomical features of the RV prevent accurate assessment of global contractility using a single index. (2) Loading conditions may also affect the assessment of RV function. RV radial function and TA motion are usually accentuated in the early and compensated stages of severe TR, and may eventually result in overestimation of RV performance. (3) Non-invasive (i.e., echocardiography-derived) measurement of pulmonary arterial pressure can be misleading when TR severely alters the pressure dynamics between the right heart chambers, reducing the reliability and accuracy of TR maximal velocity for prediction of pulmonary systolic pressure. This effect may also significantly impact the assessment of RV–pulmonary artery coupling. Therefore, caution is needed to prevent underestimation of pulmonary pressure using echocardiography. (4) TR may develop after cardiac surgery, particularly that involving the mitral valve, and RV longitudinal function is typically reduced in this setting, even when overall RV function is preserved. RV longitudinal function parameters should be used with caution in this setting.

As a result of the abovementioned limitations, no single measure offers perfect diagnostic and prognostic accuracy for assessment of RV function in the setting of TR and a multiparametric evaluation is usually recommended [12].

Mitral regurgitation frequently coexists with TR. Mitral regurgitation may occur as a primary left-sided valvular abnormality or may develop secondary to left-sided cardiac pathology (e.g., dilated cardiomyopathy or ischemic heart disease); both etiologies can contribute to VSTR. Alternatively, mitral regurgitation and TR may occur concomitantly as part of a secondary atrial mechanism driven by atrial fibrillation or heart failure with preserved ejection fraction. The presence, etiology, and severity of mitral regurgitation can influence TR severity and RV function, and vice versa, and must therefore be systematically integrated into the comprehensive echocardiographic and clinical evaluation. Following mitral valve intervention, progression of TR is commonly observed and has been associated with adverse long-term outcomes [30, 31]. Consequently, current guidelines recommend concomitant TV surgery in patients with at least moderate TR undergoing left-sided valve procedures [12]. The effect of a TV intervention on mitral regurgitation is less well defined. Cannata *et al*. [32] reported that the severity of mitral regurgitation varied considerably after transcatheter TV intervention, with mitral regurgitation remaining stable in 61% of patients, worsening in 10%, and improving in 30%. Additional studies are required to clarify the bidirectional interaction between mitral regurgitation and TR and its therapeutic implications.

## 5. Echocardiographic Assessment of RV Function

**Fig. 3.**
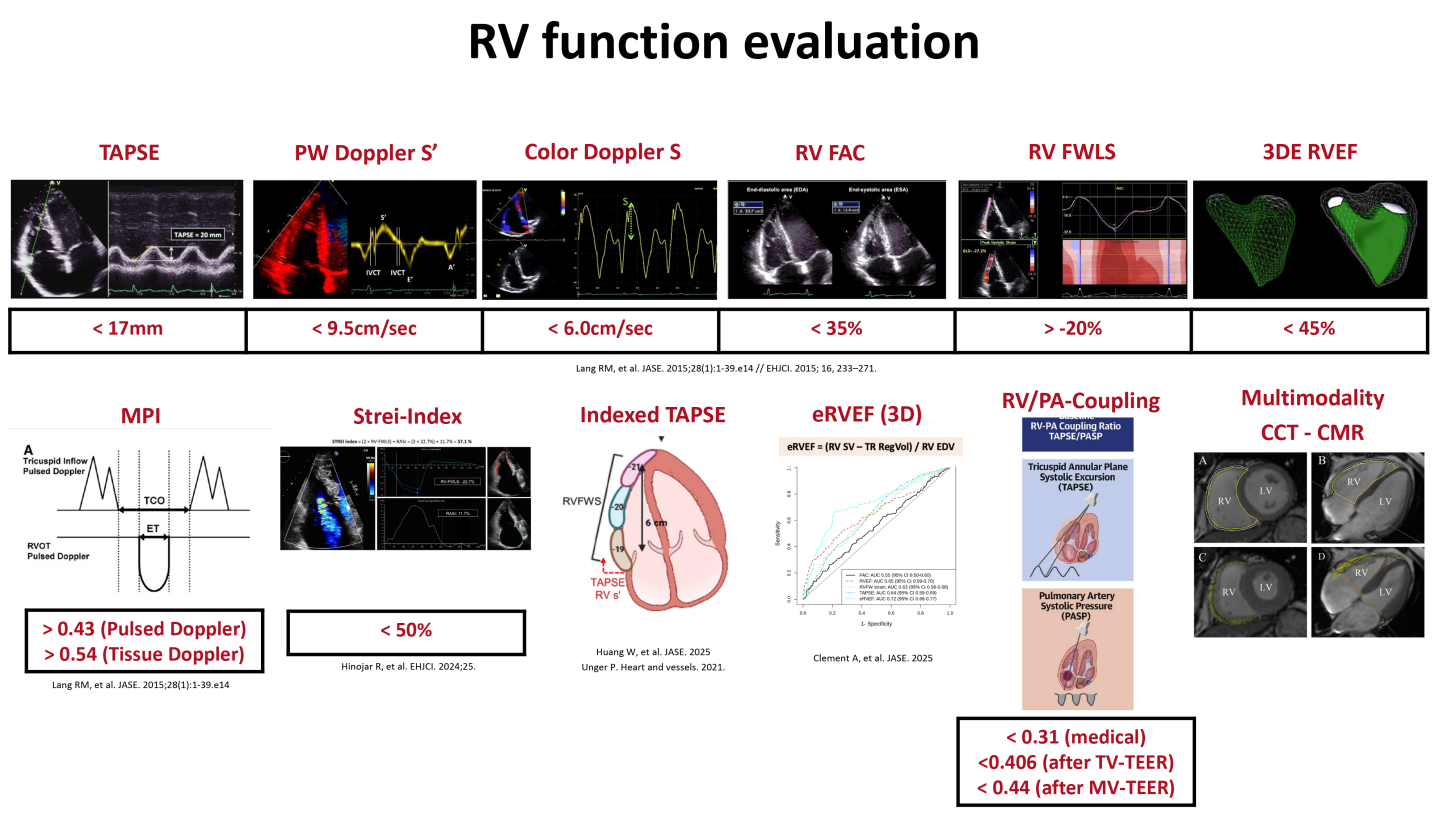
**Assessment of right ventricular function using different parameters from echocardiography, cardiac magnetic resonance imaging, or cardiac computed tomography**. Abbreviations: 3DE, three-dimensional echocardiography; CCT, cardiac computed tomography; CMR, cardiac magnetic resonance; EF, ejection fraction; eRVEF, effective RVEF; FAC, fractional area change; FWLS, free wall longitudinal strain; MPI, myocardial performance index; MV, mitral valve; PA, pulmonary artery; PW, pulsed wave Doppler; RV, right ventricle; RVEF, right ventricular ejection fraction; TAPSE, tricuspid annular plane systolic excursion; TEER, transcatheter edge-to-edge repair; TV, tricuspid valve.

Echocardiography is the first-line imaging modality for assessing TV anatomy, the size and function of the RA and RV, and quantifying TR severity [33, 34, 35, 36]. However, echocardiographic evaluation of the right heart remains challenging due to the abovementioned limitations, including the complex crescentic three-dimensional geometry of the RV and the hemodynamic impact of TR itself [34, 35]. Fortunately, advances in three-dimensional echocardiography and other imaging modalities have enhanced our ability to assess RV function. Fig. 3 provides an overview of parameters for RV function assessment using the different imaging modalities.

The echocardiographic assessment should start with a qualitative evaluation of RV anatomy using multiple acoustic windows, including the parasternal long-axis and RV inflow views, an RV-focused apical four-chamber view, and the subcostal four-chamber view. A multiparametric evaluation then provides a comprehensive assessment of RV systolic function. Table 1 provides an overview of the many echocardiographic parameters available, with their advantages and limitations. Ideally, several of the echocardiographic parameters should be used, including tricuspid annular plane systolic excursion (TAPSE), RV fractional area change (FAC), tissue Doppler-derived systolic velocity of the lateral TA (S^′^), RV global longitudinal strain (GLS), RV free wall longitudinal strain (FWLS), and three-dimensional echocardiographic RVEF [34, 37].

**Table 1.** **Overview of the echocardiographic parameters for the assessment of RV function**.

Echocardiographic parameter	Recommended method	Advantages	Limitations
Global RV function			
Pulsed wave Doppler RIMP (Tei-index)	RIMP = (TCO – ET)/ET	∙ Less influenced by heart rate	∙ Unreliable when RA pressures are elevated
		∙ Less dependent on image quality	
Tissue Doppler RIMP	RIMP = (TCO – ET)/ET	∙ Less influenced by heart rate	∙ Unreliable when RA pressures are elevated
		∙ Less dependent on image quality	
FAC	Percentage change of end-diastolic and end-systolic area of the RV, measured on an RV-focused apical view FAC (%) = 100 × (EDA – ESA)/EDA	∙ Reflects both longitudinal and radial contraction	∙ Disregards the contribution of the RV outflow tract to global RV function
		∙ Correlates with EF measured by CMR	∙ Poor reproducibility
		∙ Prognostic value in TR	∙ Load-dependent
			∙ Dependent on good image quality for endocardial delineation
Myocardial work	Using left-sided software for the evaluation of myocardial work on the RV, using RV GLS and the invasively acquired systolic and diastolic pulmonary artery pressures	∙ Assessment of RV contractility, accounting for RV after-load and dyssynchrony	∙ Inapplicable in patients with no/suboptimal TR Doppler signal
			∙ All limitations of 2DE longitudinal strain apply
Volumetric assessment	Percentage change of end-diastolic and end-systolic volume of the RV, measured by 3DE	∙ Provides additive prognostic information in specific patient populations	∙ Requires dedicated 3DE software, and consequently limited availability
	RVEF = (RVEDV – RVESV)/RV EDV × 100%		∙ Dependent on good image quality
			∙ Load dependency
3DE RV ejection fraction	Dedicated 3DE-software calculation of the RV EF	∙ Includes RV outflow tract contribution to overall function	∙ Requires dedicated 3DE software, and consequently limited availability
		∙ Independent of geometric assumptions	∙ Dependent on good image quality
		∙ Correlates well with RV EF assessed by CMR	∙ Load dependency
		∙ Established prognostic value, superior to other RV parameters	
Effective 3DE RVEF	Takes into account the volume overload	∙ All the pros of 3DE RVEF	∙ All the cons of 3DE RVEF
	eRVEF = (RV forward SV)/(RVEDV)		∙ Requires accurate calculation of the regurgitant volume
Longitudinal RV function			
TAPSE	Longitudinal lateral annular excursion M-mode, measured between end-diastole and peak systole	∙ Easy, widely available	∙ Only representative for basal lateral function
		∙ Heart rate independent	∙ Angle- and load-dependent
		∙ Reproducible	∙ Not advised in post-cardiac surgery patients
		∙ Validated against nuclear ejection fraction	
		∙ Established prognostic value	
Pulsed wave tissue Doppler S^′^	Peak systolic velocity of the lateral tricuspid annulus by pulsed wave tissue Doppler imaging	∙ Easy	∙ Only representative for basal lateral function
		∙ Good reproducibility	∙ Angle- and load-dependent
		∙ Validated against nuclear ejection fraction	
Global longitudinal strain	Longitudinal speckle tracking derived strain, averaged over the six segments of the RV	∙ Less angle- and load-dependent	∙ Vendor-dependent values
		∙ Reproducible	∙ Requires post-processing
		∙ Established prognostic value, additive to other RV parameters	∙ Requires good image quality
Free wall longitudinal strain	Longitudinal speckle tracking derived strain, averaged over the three segments of the lateral free wall of the RV	∙ Less angle- and load-dependent	∙ Vendor-dependent values
		∙ Reproducible	∙ Requires post-processing
		∙ Established prognostic value, additive to other RV parameters	∙ Requires good image quality
RV-PA-coupling			
TAPSE/sPAP	The ratio between TAPSE and echocardiographic estimated sPAP	∙ Reflects RV contractility, accounting for load-dependence	∙ Limited accuracy due to non-invasive sPAP measurements
RVFWLS/sPAP	The ratio between RVFWLS and echocardiographic estimated sPAP	∙ Reflects RV contractility, accounting for load-dependence	∙ Limited accuracy due to non-invasive sPAP measurements
Novel methods			
Forward SV/RV ESV	Ratio between the calculated forward SV and 3DE-aquired RV ESV	∙ All the pros of the volumetric assessment (3DE)	∙ All the limitations of 3DE RVEF
			∙ Requires accurate calculation of the regurgitant volume
RV free wall longitudinal fractional shortening	Indexed TAPSE to be used as a surrogate for RV free wall longitudinal strain = TAPSE/RVdiastolic length	∙ Easy	∙ RV diastolic length is dependent on the degree of RV maladaptive remodeling
		∙ No need for dedicated software	
		∙ Excellent intraclass correlation coefficients for inter- and intra-observer variability	

Abbreviations: 2DE, two-dimensional echocardiography; 3DE, three-dimensional echocardiography; CMR, cardiac magnetic resonance; EDA, end-diastolic area; EDV, end-diastolic volume; EF, ejection fraction; eRVEF, estimated right ventricular ejection fraction; ESA, end-systolic area; ESV, end-systolic volume; ET, ejection time; FAC, fractional area change; FWLS, free wall longitudinal strain; GLS, global longitudinal strain; RA, right atrium; RIMP, Right Ventricular Index of Myocardial Performance; RV, right ventricle; RVFWLS, right ventricular free wall longitudinal strain; sPAP, systolic pulmonary artery pressure; SV, stroke volume; TAPSE, tricuspid annular plane systolic excursion; TCO, total contraction time; TR, tricuspid regurgitation.

New measures have been developed to optimize RV function assessment by eliminating some of the limitations of conventional parameters. In this context, the effective RVEF accounts for the regurgitant volume in the presence of TR by calculating the ratio between the RV forward stroke volume and the RV end-diastolic volume [38]. In addition, measures assessing the RVPAC account for pulmonary arterial afterload, offering a more physiologically integrated assessment of RV function than conventional deformation or volumetric parameters alone, and reflecting the adequacy of RV adaptation to pressure overload [39, 40]. RV function and pulmonary pressure are closely linked because PHT can impair RV function and, conversely, severe RV dysfunction can prevent the generation of elevated pulmonary pressures. RVPAC is traditionally defined as the ratio of end-systolic ventricular elastance to arterial elastance as measured using invasive right heart catheterization. However, echocardiography provides a non-invasive surrogate by calculating the ratio of a longitudinal parameter (TAPSE or RVFWLS) to the systolic pulmonary arterial pressure (sPAP) estimated by echocardiography. This load-adjusted metric may enhance risk stratification and guide timing of intervention in conditions where afterload is elevated and conventional measures may underestimate RV dysfunction. In patients with significant TR, a reduced TAPSE/sPAP ratio consistently emerges as a marker of RV–pulmonary arterial uncoupling and adverse prognosis. The optimal prognostic threshold is context-dependent, with reported values of approximately 0.31 in STR [41, 42], 0.40 in isolated functional TR [43], 0.406 in transcatheter TR intervention cohorts [44], and up to 0.49 in a mixed TR population [45]. A threshold of 0.31 mm/mmHg is most frequently associated with excess mortality in patients with STR. Taken together, these data support a continuum in which TAPSE/sPAP values less than ~0.3–0.4 mm/mmHg indicate clinically relevant RV–pulmonary artery (PA) uncoupling and identify higher-risk patients, whereas higher values are more compatible with preserved coupling. Furthermore, the pulmonary artery pulsatility index is a hemodynamic measure of RVPAC, calculated as (sPAP – pulmonary artery diastolic pressure) divided by right atrial pressure. In patients with significant TR, available data suggest a largely continuous inverse relationship between the pulsatility index and outcome, without a universally accepted TR-specific cutoff value. In patients undergoing transcatheter TV intervention, who were stratified according to pulsatility index values (<2, 2–4, and >4) indicates that values <2 identified the highest-risk patients, whereas higher values (≥3–4) were more consistent with relatively preserved RVPAC, although these thresholds remain exploratory rather than guideline-endorsed [46]. Lastly, it has been suggested that RV longitudinal fractional shortening can account for the influence of diastolic RV length on TAPSE, providing an adjusted metric that serves as a practical surrogate for RV FWLS. Clinically, this parameter may offer a simple yet physiologically relevant means to evaluate RV longitudinal function, particularly in settings where strain imaging is not readily available [47].

Most Doppler methods used to quantify left-sided valvular regurgitation can be used to assess TR severity. This includes jet size, vena contracta width, and proximal convergence analysis. However, some characteristics of TR jets are inherently different from mitral regurgitation jets. Importantly, except in severe PHT, the TR jet is usually characterized by a lower pressure gradient (and thus lower velocity) than in mitral regurgitation, as a result of the lower RV systolic pressures. This difference may significantly impact jet analysis [48]. Jet flow, and consequently color Doppler jet area, is mainly driven by conservation of momentum (flow × velocity = EROA × [Velocity]^2^). This implies that for a similar EROA, the color jet area of a TR jet, reaching half the velocity of a mitral regurgitation jet, will be 4 times smaller than the mitral regurgitation jet, provided that machine settings are similar (including gain, color Doppler scale, and aliasing velocity). In addition, since RA size correlates with TR severity, the use of a fixed ratio of jet size/RA area size would tend to underestimate TR severity. The TR regurgitant orifice is usually non-circular and even nonplanar, which may lead to highly variable vena contracta measurements depending on the imaging plane. When TR is severe, RA pressure may rise in early systole with early velocity peaking, the continuous wave spectral shape is dense and triangular, and the peak jet velocity is frequently low (<2.5 m/s). Quantitative assessment of TR by the proximal isovelocity surface area (PISA) method may also have limitations. Indeed, the lower the maximal velocity, the larger the underestimation of flow, as the contours of the regurgitant isovelocities flatten [49]. In addition, the regurgitant orifice in STR is often ellipsoid and the resulting PISA becomes more hemi-elliptical rather than hemispheric [50]. Despite these known limitations and pitfalls, echocardiography remains central to the assessment of TR severity in routine clinical practice [12].

## 6. Multi-Modality Imaging for the Assessment of RV function

### 6.1 Cardiac Magnetic Resonance Imaging

Cardiac magnetic resonance (CMR) imaging is currently the gold standard for quantifying RV size and systolic function due to its high spatial resolution and independence from geometric assumptions [51]. Moreover, CMR provides detailed tissue characterization using late gadolinium enhancement and extracellular volume mapping, identifying myocardial scar/fibrosis or infiltrative processes that impact RV function [52]. Recent advances in CMR enable strain-imaging by CMR-derived feature tracking during post-processing, which appears to be an early marker of RV dysfunction [53]. However, despite its well-established advantages in providing detailed and reproducible assessments of RV function, CMR imaging has limitations that hinder widespread clinical adoption. These include limited availability, prolonged acquisition and post-processing times, and higher costs compared to other imaging modalities. A recent survey by the European Association of Cardiovascular Imaging revealed the magnitude of the impact of these limitations: only 7.25% of respondents reported using CMR as the primary imaging modality for assessment of RV function [54].

### 6.2 Cardiac Computed Tomography

Cardiac computed tomography (CCT) is not routinely used for the assessment of RV function because of its limited availability, exposure to ionizing radiation, and need for iodinated contrast agents, in particular in patients with significant TR, which can lead to end-organ damage including cardio-renal syndrome. However, CCT offers high three-dimensional spatial resolution, enabling unlimited multiplanar reformats and providing a detailed visualization of the entire right heart, including the RV outflow tract and pulmonary arteries, with an accurate quantification of RV volumes and TA dimensions [55, 56]. A detailed anatomical assessment of the right heart structures, in particular the TA and the surrounding tissues, is crucial when planning transcatheter TV interventions. This requirement has led to increased use of CCT in symptomatic patients with significant TR. In addition to providing good anatomical resolution, RV volumes and EF values derived from CCT have been shown to be strongly correlated with those obtained using CMR. In a study by Tanaka *et al*. [57], CCT was used to evaluate RVEF in symptomatic patients with severe TR who were undergoing transcatheter TV repair. CCT-derived RV functional parameters provided additional prognostic information beyond that of conventional echocardiographic indices, highlighting the potential role of CCT in the comprehensive pre-procedural evaluation and risk stratification of this patient population.

### 6.3 Other Imaging Modalities

Historically, nuclear imaging techniques have been the most widely used method for assessing RV function. These techniques provide accurate quantification of RV volumes and ejection fraction, derived from differences in end-diastole and end-systole radionuclide count densities, thereby eliminating the need for the geometric assumptions inherent to other imaging modalities [58, 59]. First-pass and equilibrium radionuclide ventriculography techniques have been extensively validated for this purpose [60, 61]. However, despite some diagnostic robustness, nuclear methods are limited by relatively low temporal resolution and risks associated with exposure to ionizing radiation. In the current era dominated by 3D echocardiography and CMR, the main advantage of nuclear imaging is its ability to provide additional insights into myocardial perfusion and metabolic activity [62].

## 7. Invasive Hemodynamic Assessment

Echo Doppler is the primary imaging technique for assessing TR and guiding management decisions. However, massive or torrential TR may hinder the non-invasive assessment of sPAP, because the Bernoulli equation is not applicable in such conditions and right atrial pressure is markedly elevated. This effect leads mainly to an underestimation of sPAP when compared with invasive measurements [63, 64]. In a series of 243 patients undergoing transcatheter TV repair, RV systolic pressure values obtained non-invasively and by right heart catheterization were discordant in 23% of the patients, and a discordant pattern with invasive sPAP ≥50 mmHg and echo values <50 mmHg independently predicted death, heart failure hospitalization, and reintervention [65]. Direct pressure measurement via right heart catheterization may therefore be required to resolve uncertainty, particularly if values obtained from transthoracic echocardiography remain questionable or inconsistent with clinical data. Moreover, wedge pressure, which is critical to determine the pre- or post-capillary predominance of PHT, is not reliably assessed using echocardiography. Patients with pre-capillary PHT are considered at high risk and have been largely excluded from randomized trials on percutaneous TV intervention. These factors further highlight the important role of right heart catheterization, which may, in addition, play a role in optimization of heart failure therapy. RVPAC is an important prognostic marker in TR [66]. Data from the EuroTR investigators showed an improved prognostic value of RVPAC in patients undergoing transcatheter tricuspid valve edge-to-edge repair (T-TEER) when sPAP was directly assessed using right heart catheterization (TAPSE/sPAP cutoff 0.303 mm/mmHg) compared to echo-derived sPAP assessment (cutoff 0.387 mm/mmHg) [67]. Thus, given the prognostic implications, a precise pulmonary hemodynamic assessment is mandatory in TR, providing crucial information that can influence the type and timing of intervention. The 2020 AHA/ACC guideline for the management of patients with valvular disease gives a class IIa recommendation for right heart catheterization in patients with TR if clinical and non-invasive data are considered discordant or inadequate [13]. The 2025 ESC guidelines recommend right heart catheterization in all candidates for intervention, to assess the hemodynamic consequences of TR on the right atrium and venous circulation, measure end-diastolic RV pressure, and document volume overload [12].

## 8. RV Function Assessment and Association With Outcome in Patients With Significant TR

RV dysfunction in the setting of significant TR independently predicts poor outcomes, including all-cause mortality, heart failure–related hospitalizations, and reduced functional capacity. However, large-scale studies validating the prognostic implications of RV dysfunction in this context are limited [17]. Table 2 (Ref. [9, 18, 38, 41, 44, 53, 57, 67, 68, 69, 70, 71, 72, 73, 74, 75, 76, 77, 78, 79, 80, 81, 82, 83, 84, 85, 86, 87, 88, 89, 90, 91, 92]), provides an overview of the studies that have assessed the association of different RV function parameters with outcome. In medically treated patients with significant TR, approximately two-thirds exhibit RV dysfunction, which is commonly defined as a TAPSE <17 mm. This finding correlates with poorer outcomes, even in the absence of significant RV dilation [68]. Moreover, pre-procedural RV dysfunction, defined as low TAPSE (TAPSE <17 mm) and reduced RVFAC (FAC <35%), also predicted worse outcomes following surgical or transcatheter TV intervention [9, 69]. In transcatheter TV interventions, favorable outcomes are more likely when longitudinal impairment is offset by preserved or enhanced circumferential function, whereas dual impairment in both domains is associated with poor prognosis [70].

**Table 2.** **Overview of published studies assessing the association of different RV function parameters with outcome**.

Parameter for RV-function assessment	Population	Outcome
Conventional echocardiographic parameters: TAPSE, S^′^, FAC		
Dietz MF, *et al*. [82]	1311 patients with significant (≥ moderate) secondary TR, medically treated	∙ Stages of right HF were independently associated with all-cause mortality at long-term follow-up.
Dietz MF., *et al*. [68]	1292 patients with significant (≥ moderate) secondary TR	∙ The 5-year survival rate was significantly worse in patients presenting with RV dysfunction (assessed by TAPSE) in comparison with normal RV function.
		∙ RV dysfunction was independently associated with poor outcome on multivariable analysis.
Zornitzki L., *et al*. [83]	1143 patients with significant (≥ moderate) TR	∙ TAPSE <18.0 mm and S^′^ <10.0 cm/sec were the cutoffs associated with excess mortality.
		∙ The TAPSE and S^′^ cutoffs associated with excess mortality were lower in patients with significant TR compared to patients without.
		∙ In a multivariate model, TAPSE and S^′^ were independently associated with mortality.
Galloo X., *et al*. [9]	278 patients with significant TR undergoing TV surgery	∙ Patients with a more advanced stage of right HF had worse survival.
		∙ A less severe stage of right HF was independently associated with better survival.
Vogelhuber J., *et al*. [69]	262 patients with symptomatic TR undergoing TEER	∙ RV dysfunction before TEER was associated with an increased risk of all-cause and cardiovascular death, and hospitalization due to HF during follow-up.
		∙ The worse outcomes were mainly attributable to impaired global RV function.
Rodríguez Torres DJ., *et al*. [18]	70 patients undergoing cardiac and TV surgery	∙ No relationship between RV function parameters and mortality or major complications after TV surgery.
2D Speckle tracking echocardiography: RV GLS and RV FWLS		
Prihadi E., *et al*. [71]	896 patients with significant (≥ moderate) secondary TR	∙ Non-survivors had worse RV systolic dysfunction.
		∙ Cumulative event-free survival was significantly worse in patients with decreased RV function.
		∙ On multivariate analysis, RV FWLS was independently associated with all-cause mortality and incremental to FAC and TAPSE.
Ogawa M., *et al*. [84]	53 patients with severe atrial secondary TR associated with atrial fibrillation	∙ In multivariable analysis, reduced RV FWLS was independently associated with all-cause death.
		∙ Patients with RV FWLS ≤18% had higher risk of all-cause death adjusted for age.
Ogawa M., *et al*. [72]	377 patients with severe secondary TR	∙ RVFWLS provided better prognostic information than RV FAC by ROC curve analysis.
		∙ In the multivariable Cox regression analysis, elevated right atrial pressure and RVFWLS of ≤18% were independent predictors of clinical outcome.
Curtis E., *et al*. [73]	262 consecutive patients undergoing echocardiography and right-heart catheterization on the same day.	∙ Preserved RV FWLS was correlated with better outcomes, although this was only statistically significant in patients without severe TR or PHT.
		∙ Abnormal RV FWLS to pulmonary pressures and RV size ratios were significantly correlated with adverse outcomes.
Hinojar R., *et al*. [74]	151 patients with severe secondary TR and no formal indication for TV intervention.	∙ 35% of the patients reached the combined end point.
		∙ Cumulative event-free survival was significantly worse in patients with impaired RV GLS and RV FWLS.
		∙ Conventional indices of RV systolic function were not associated with outcomes.
		∙ In multivariate analysis, RV FWLS was independently associated with mortality and HF.
Ancona F., *et al*. [85]	79 consecutive patients with severe TR undergoing isolated TV surgery	∙ RVFWLS was the best parameter to predict peri-operative mortality.
		∙ The combination of TRI-SCORE and RVFWLS outperformed classic TRI-SCORE in outcome prediction.
Kim M., *et al*. [86]	115 patients with severe secondary TR who underwent isolated TV surgery	∙ An absolute preoperative RVFWLS <24% was associated with the primary end-point, independent of clinical risk factors.
		∙ Other conventional echocardiographic measures of RV function were not significant.
3D Echocardiography		
Tomaselli M., *et al*. [87]	554 patients with moderate and severe secondary TR, under medical treatment	∙ Men and women had the same incidence of all-cause mortality and HF hospitalization.
		∙ Women and men had similar risk at lower EROAs, smaller regurgitant volume, smaller dimensions, and higher RVEF.
Ladányi Z., *et al*. [75]	205 consecutive adult patients referred for echocardiography with secondary TR	∙ RV mechanics and global function change at different stages of TR severity.
		∙ The relative contribution of radial shortening was independently associated with the combined endpoint of all-cause death and HF hospitalization, whereas conventional RV functional measures, including RVEF, were not.
Badano L., *et al*. [88]	758 patients with moderate-to-severe secondary TR	∙ 3 phenogroups of RV remodeling were identified:
		∘ Low-risk phenogroup: moderate TR, preserved RV size and function, and a moderately dilated but normally functioning right atrium.
		∘ Intermediate-risk phenogroup: older patients with severe TR, and a mildly dilated but uncoupled RV.
		∘ High-risk phenogroup: younger patients with massive-to-torrential TR, severely dilated and dysfunctional RV and right atrium.
		∙ Multivariable analysis confirmed the clustering as independently associated with the composite endpoint.
Tomaselli M., *et al*. [76]	513 patients with moderate and severe secondary TR	∙ EROA independently predicted outcomes in secondary TR.
Formula: EROA corrected for PISA		∙ An EROAc >0.47 cm^2^ was associated with a 2-fold increased risk (high-risk patient).
EROAc = 6.28 × r^2^ × V_a_ × (α/180) × (V_p_/[V_p_–V_a_])		∙ For low-risk patients with EROAc ≤0.47 cm^2^, evaluating RV function and RV-pulmonary artery coupling enhanced risk stratification.
Clement A., *et al*. [38] Formula eRVEF = RV forward SV/RV ESV	513 patients with first echocardiographic diagnosis of mild to severe secondary TR	∙ Time-dependent ROC analysis showed a stronger association with outcome for eRVEF than ‘normal’ RVEF, TAPSE, RV FWLS and RV FAC.
		∙ The eRVEF cutoff associated with an excess event rate was 20% on spline curve modeling.
		∙ In multivariable analysis, eRVEF as a continuous variable remained independently associated with the combined endpoint.
Orban M., *et al*. [89]	75 patients with severe TR undergoing TV-TEER	∙ Impaired preprocedural 3D-RVEF was associated with mortality after TTVR, but the postprocedural decrease in 3D-RVEF after TTVR was not.
Multi-modality imaging		
1. Cardiac magnetic resonance		
Hinojar R., *et al*. [77]	75 patients with significant TR (≥ severe) undergoing a CMR study	∙ 39% experienced the endpoint.
		∙ After adjustment, both eRVEF ≤34% and RV shortening ≥–14% were significantly associated with outcomes.
		∙ Among all parameters of RV function, effective RVEF was the strongest predictor of outcomes, incremental to RVEF.
Romano S., *et al*. [53]	544 consecutive patients with severe secondary TR undergoing CMR	∙ RV FWLS ≥ median (–16%) had significantly reduced event-free survival.
		∙ By Cox multivariable regression modeling, RV FWLS was associated with increased risk-of-death after adjustment for clinical and imaging risk factors.
Park JB., *et al*. [90]	75 patients with severe secondary TR	∙ Cardiac death risk was lower with a higher RVEF.
		∙ On multivariable analysis, RVEF remained a significant predictor for cardiac death and major postoperative cardiac events.
		∙ RV ESV index was independently associated with outcomes.
Kresoja KP., *et al*. [70]	79 patients with severe TR undergoing TTVR	∙ Global RV dysfunction but not longitudinal RV dysfunction was a predictor of outcomes among TTVR patients.
		∙ 3 patterns of RV contraction, in which a loss of longitudinal function can be compensated by increasing circumferential function, preserving RVEF and favorable outcomes.
2. Cardiac Computed Tomography		
Tanaka T., *et al*. [57]	157 symptomatic patients with TR who underwent CCT before TTVR	∙ CT-RVEF <45% was associated with a higher risk of the composite outcome.
		∙ CT-RVEF had an additional value beyond 2D echocardiographic assessment of RV-function.
Kirchner J., *et al*. [91]	100 patients with severe TR undergoing TTVR	∙ At 1 year the primary endpoint occurred significantly more in patients with RV EF <50% (36.6% vs. 13.7%).
		∙ Patients with dysfunctional RVs demonstrated worse outcome than patients with functional RVs (43.7% vs. 12.2%).
Novel measures of RV function		
1. Echocardiographic RV-PA coupling		
1.1. TAPSE/sPAP		
Fortuni F., *et al*. [41]	1149 patients with ≥ moderate secondary TR	∙ The cumulative 5-year survival rate was lower in patients with RV-PA uncoupling compared to their counterparts (37% vs. 64%).
		∙ After correcting for potential confounders, RV-PA uncoupling was the only echocardiographic parameter independently associated with all-cause mortality.
Brener M., *et al*. [44]	444 patients undergoing transcatheter TV intervention	∙ TAPSE/sPAP ratio >0.406 was associated with a decreased risk of all-cause mortality.
Stolz L., *et al*. [67]	848 patients who underwent TV-TEER	∙ Uncoupling assessed by echocardiography as well as invasively predicts 2-year all-cause mortality, however signicantly higher c-index was observed when using the invasive assessment.
Sugiura A., *et al*. [92]	206 patients who underwent TV-TEER	∙ Invasive assessment of RV-coupling was inversely associated with all-cause mortality or HF hospitalization within 1year after the procedure.
1.2. TAPSE/RVFWLS		
Ancona F., *et al*. [78]	250 consecutive patients with severe TR	∙ RV FWLS/sPAP ≤0.34%/mmHg was associated with baseline clinical RV failure.
		∙ RV FWLS/sPAP, but not TAPSE/sPAP, was independently correlated with all-cause mortality.
1.3. 3DE-derivde RV-PA-coupling		
Gavazzoni M., *et al*. [79]	108 patients with moderate or severe secondary TR	∙ RV forward SV/ESV is associated with the risk for death and heart failure hospitalization in patients with STR.
Formula = RV forward SV/RV ESV		∙ A RV forward SV/ESV ratio <0.4 is associated with higher related risk.
2. STREI-index		
Hinojar R., *et al*. [80]	176 consecutive patients with isolated ≥ severe TR	∙ Identified a higher percentage of patients with RV dysfunction compared with conventional parameters.
Formula = [2 × (RVFWLS)] + RASr		∙ Predicted CV events, independently of TR severity and RV dimensions.
3. RV Contractile reserve		
Utsunomiya H., *et al*. [81]	36 patients with severe secondary TR	∙ TAPSE/sPAP slope ≤0.046 mm/mmHg was independently associated with all-cause mortality.
		∙ The cumulative survival rate was lower in patients with TAPSE/sPAP slope ≤0.046 mm/mmHg compared with their counterparts.

Abbreviations: 2D, two-dimensional; 3D, three-dimensional; CT, cardiac computed tomography; CMR, cardiac magnetic resonance imaging; EF, ejection fraction; EROA, effective regurgitant orifice area; eRVEF, effective right ventricular ejection fraction; ESV, end-systolic volume; FAC, fractional area change; FWLS, free wall longitudinal strain; GLS, global longitudinal strain; HF, heart failure; PA, pulmonary artery; PISA, proximal isovelocity surface area; RASr, right atrial reservoir strain; ROC, receiver operating characteristic; RV, right ventricle; S’, tissue Doppler imaging S’; sPAP, systolic pulmonary artery pressure; SV, stroke volume; TAPSE, tricuspid annular plane systolic excursion; TEER, transcatheter edge-to-edge repair; TTVR, transcatheter tricuspid valve repair; TV, tricuspid valve.

Among patients with STR, RVFWLS identifies RV dysfunction in approximately 85% of patients (versus 72% by TAPSE, and 49% by FAC) [71]. RVFWLS independently predicts all-cause mortality and provides additional prognostic value beyond that of TAPSE, FAC, and TR severity [71, 72, 73, 74]. Reduced 3DE-derived RVEF is associated with higher mortality and cardiac death across various cardiovascular disease cohorts [8, 93]. In STR, 3DE reveals significant RV remodeling and different contraction patterns as TR severity increases, with a decline in longitudinal shortening, whereas radial and anteroposterior contractions remain stable. Radial shortening, in turn, correlates with prognosis [75]. Furthermore, assessment of effective RVEF (which takes regurgitant volume into account) using 3DE has a stronger association with mortality and heart failure hospitalization than standard 3DE-derived RVEF [76].

Multimodality imaging further improves prognostic accuracy. In prospective CMR studies of severe TR, effective RVEF and feature-tracking-derived RVFWLS independently predicted death, in addition to clinical and other imaging risk factors [53, 77]. Similarly, CT-derived RVEF (<45%) is associated with poor outcomes following TV interventions [57]. New metrics, such as RV–pulmonary artery coupling [41, 78, 79], strain-based composite indices (e.g., STREI) [80], and contractile reserve during stress [81], offer additional prognostic discrimination. These metrics may refine the choice and timing of interventions for patients with significant TR.

## 9. Integrating RV Function Assessment into Clinical Decision Making

**Fig. 4.**
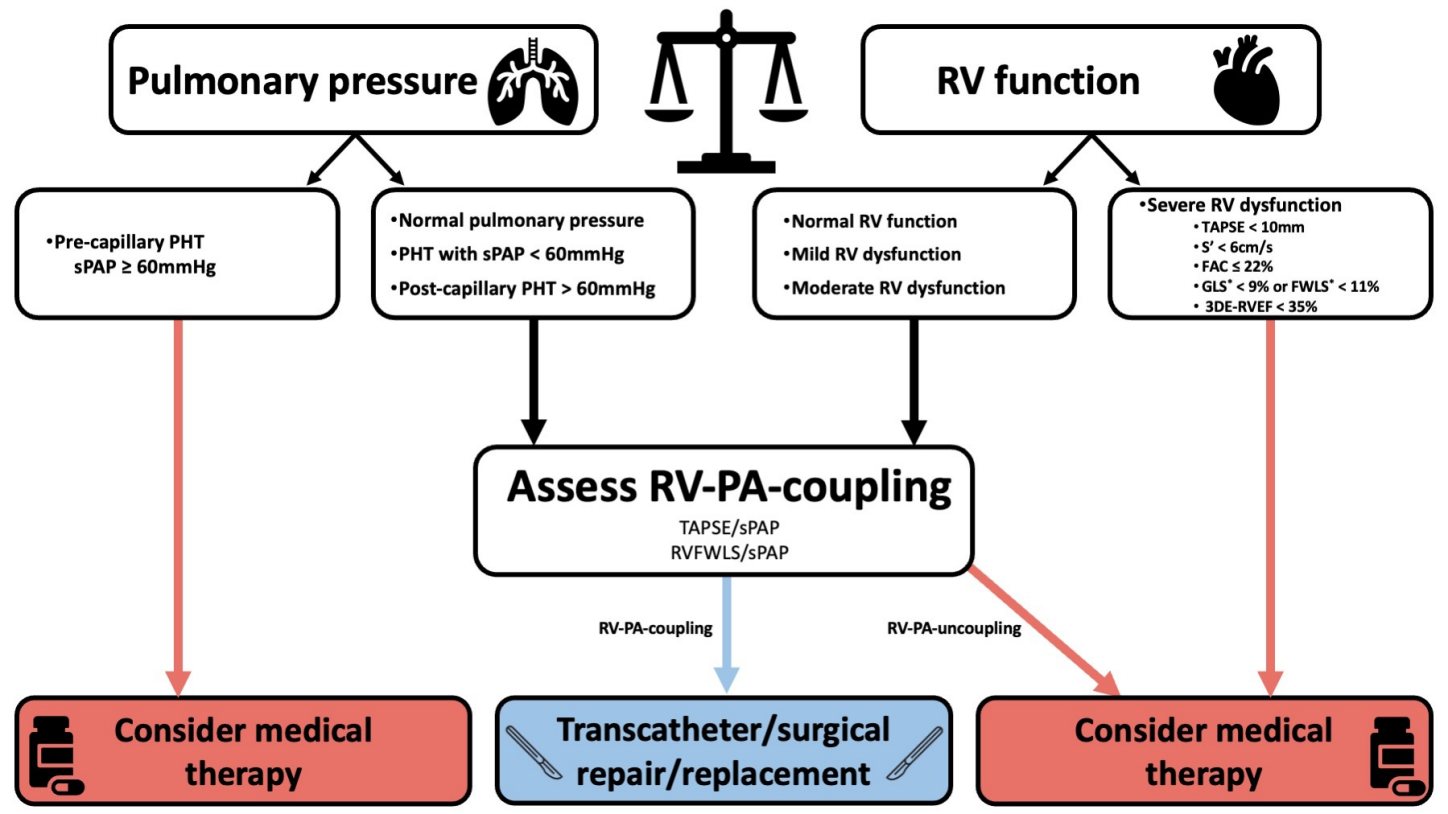
**Proposed algorithm for clinical decision making integrating the assessment of pulmonary pressure and RV function**. *expressed as absolute (i.e., positive) values. The exact cutoffs to define RV-PA-uncoupling may vary according to the selected patient population (these values are discussed in chapter 5 - Echocardiographic Assessment of RV Function). Abbreviations: 3DE, three-dimensional echocardiography; FAC, fractional area change; FWLS, free wall longitudinal strain; GLS, global longitudinal strain; PHT, pulmonary hypertension; RV, right ventricle; RVEF, right ventricular ejection fraction; RV-PA-coupling, right ventricular to pulmonary artery coupling; sPAP, systolic pulmonary arterial pressure; TAPSE, tricuspid annular plane systolic excursion.

Decision-making in the management of TR involves the assessment of various factors, including patient-related, anatomical, hemodynamic, and RV function aspects. Patient-related factors encompass comorbidities, age and life expectancy, quality of life, and rehabilitation capacity. Anatomical factors involve the determination of primary vs secondary vs cardiac implantable electronic device-related-TR, leaflet morphology, and the location of the jet. Because echocardiography often underestimates pulmonary pressures in the presence of significant TR, invasive assessment of pulmonary arterial pressure as an estimate of RV afterload and of pulmonary vascular resistance to rule out precapillary PHT is mandatory. Fig. 4 shows an algorithm guiding clinical decision making for medical therapy or surgical TV intervention, based on the assessment of pulmonary pressure and RV function.

Elevated pre-capillary pulmonary arterial systolic pressure >60 mmHg [94] or >70 mmHg [95] has been considered a contraindication to transcatheter interventions. Importantly, the assessment of RV function should be integrated into the decision making process. Severe impairment of RV function (Table 3, Ref. [12]) has been widely used as an exclusion criterion in studies on transcatheter valve interventions, to prevent procedures with limited therapeutic value. However, the exact thresholds for RV function that should be used in the setting of transcatheter TV interventions have not been prospectively validated. Finally, among patients who have normal pulmonary pressures or post-capillary PHT, the assessment of RV-PA coupling may provide important insight. This measurement may help verify whether PAP is abnormal just because the dysfunctional RV is unable to generate high pressures or, in the case of post-capillary PHT, that RV function is compensating for the elevated pulmonary pressure. Use of the TAPSE/sPAP ratio has been proposed to identify patients who may have prognostic benefit from T-TEER compared to medical therapy. In a recent study, patients with a TAPSE/sPAP ratio of 0.32–0.46 mm/mmHg had a better survival with T-TEER than patients receiving conservative management [66]. Thus, integration of all clinical, anatomical, and functional parameters by a dedicated Heart Team is mandatory, and is listed as a Class I recommendation in current guidelines [12]. Patients undergoing left-sided valve surgery should have concomitant TV surgery if TR is severe, whether primary or secondary (Class I). In addition, concomitant TV repair should be considered in patients with moderate primary or secondary TR, to avoid TR progression and RV remodeling (Class IIa); repair may even be considered in selected patients with mild secondary TR and TA dilatation (≥40 mm or >21 mm/m^2^) (Class IIb). In symptomatic patients with severe TR, but without left-sided valvular heart disease requiring surgey, isolated TV surgery is recommended in patients without severe RV dysfunction or severe PHT (Class I), and should also be considered in: (1) asymptomatic patients with severe primary TR with RV dilatation/RV function deterioration, but without severe LV/RV dysfunction or severe PHT; and (2) patients with severe STR who are symptomatic or have RV dilatation/RV function deterioration, but without severe LV/RV dysfunction or PHT. Transcatheter TV interventions can improve quality of life and reduce RV remodeling in high-risk patients with severe TR who remain symptomatic under optimal medical therapy and do not have severe RV dysfunction or pre-capillary PHT.

**Table 3.** **Criteria for RV dysfunction assessed by echocardiography [12]**.

RV function parameter	RV dysfunction	Severe RV dysfunction
TAPSE (mm)	<17	<10
RV TDI s’ (cm/s)	<10	<6
RV GLS* (%)	<21	<9
RV FWLS* (%)	<23	<11
3D RVEF (%)	<50	<35
FAC (%)	≤35	≤22

* Values are expressed as absolute (i.e., positive) values. Abbreviations: 3D, three-dimensional; EF, ejection fraction; FAC, fractional area change; FWLS, free wall longitudinal strain; GLS, global longitudinal strain; RV, right ventricule; TAPSE, tricuspid annular plane systolic excursion; TDI, tissue Doppler imaging.

Assessing procedural risk and determining the optimal timing for referral of patients with significant TR remain challenges in clinical practice for. Despite advances in our understanding of RV and TR pathophysiology and growing experience with TV surgery, in-hospital mortality remains approximately 10% for isolated TV surgical intervention [96, 97]. Current recommendations state that patient evaluation and TV intervention should be performed in a euvolemic state, as right-sided or biventricular congestion has been associated with poorer outcomes, including lower procedural success rates and reduced survival [98]. Conventional surgical risk models, such as the European System for Cardiac Operative Risk Evaluation (EuroSCORE) [99, 100] and the Society of Thoracic Surgeons (STS) score [101], have been widely used in this context. However, the original validation cohorts of these models included a limited proportion of patients with significant TR. Consequently, these models demonstrate limited predictive accuracy in this population. Recently, several research groups have developed TR-specific risk scores for patients with severe TR who are managed medically, surgically, or with transcatheter interventions. In patients with severe TR managed conservatively, dedicated risk scores have been developed and demonstrated prognostic value; both incorporated RV function as a key variable [102, 103]. Several other risk models have been evaluated and shown to be predictive for those undergoing surgical or transcatheter TV intervention. These models include the Model for End-Stage Liver Disease (MELD) score, TRI-SCORE, TRIVALVE score, and the dedicated STS Adult Cardiac Tricuspid Valve Surgery Risk Calculator [104, 105, 106, 107, 108]. Notably, among these models, only the TRI-SCORE includes an assessment of RV function (Table 4), highlighting the limited integration of this critical parameter into existing risk stratification tools for this population. The TRI-SCORE was initially developed to predict in-hospital mortality in patients undergoing isolated TV surgery and demonstrated superior prognostic performance compared to the EuroSCORE I and II (area under the curve 81.7% vs. 66.8% and 62.9%, respectively). Based on the total score, patients can be categorized into low-, intermediate-, and high-risk strata, each associated with markedly different surgical mortality rates [105]. The TRI-SCORE was subsequently evaluated in the TRIGISTRY, a multicenter registry of 2414 patients with severe isolated secondary TR. Survival declined progressively with increasing TRI-SCORE values, irrespective of the therapeutic strategy. Moreover, among patients with low or intermediate risk, early and successful surgical or transcatheter intervention was associated with superior 2-year survival compared to conservative management [106]. More recently, the TRI-SCORE has also been validated across multiple cohorts undergoing transcatheter TV repair, further supporting its applicability and predictive value in this population [109].

**Table 4.** **The TRI-SCORE risk factors and scoring system**.

TRI-SCORE	
Risk factors	Scoring
Age ≥70 years	1
NYHA functional class III or IV	1
Right-sided heart failure signs	2
Daily dose of furosemide ≥125 mg	2
Glomerular filtration rate <30 mL/min	2
Elevated total bilirubin	2
Left ventricular ejection fraction <60%	1
Moderate/severe right ventricular dysfunction	1
FINAL TRI-SCORE	12

Abbreviations: NYHA, New York Heart Association function class.

## 10. Conclusions and Future Directions

RV function is a critical determinant and prognostic marker in patients with significant TR. Its careful assessment is mandatory for optimizing the management strategy. However, while several imaging and hemodynamic parameters have been used to characterize RV function in patients with TR, there is currently a lack of clear cutoff values and prospective validation. Further advances in the assessment of RV function will substantially improve this field, including progress in imaging technology, the integration of artificial intelligence into the daily clinical workflow, and new biomarker discovery.

Integration of novel echocardiographic parameters such as RV myocardial work [110] and segmental strain analysis holds promise for improved RV characterization and earlier detection of subclinical RV dysfunction. Additionally, CCT, particularly with newer, high-resolution, low-radiation protocols, is emerging as a viable modality for RV assessment in patients undergoing structural heart interventions [55, 111]. Furthermore, alternative metrics, such as fast-SENC intramyocardial strain, a unique CMR modality that measures intramyocardial RV contraction in 1 heartbeat per image plane, have been shown to detect subclinical RV dysfunction well before changes in RVEF; this measurement needs further clinical validation [112].

New artificial intelligence-driven algorithms are showing potential for automating and improving RV functional analysis across multiple imaging modalities. Deep learning models can segment the RV with high precision, enabling consistent quantification of RV volumes, ejection fraction, and advanced imaging modalities such as RV speckle-tracking imaging, while minimizing interobserver variability and enhancing clinical efficiency [113, 114]. Furthermore, integrating multi-parametric data from CMR, including late gadolinium enhancement, T1/T2 mapping, and feature tracking, into machine learning models may facilitate phenotypic classification and risk stratification beyond conventional metrics [115, 116].

Alongside imaging advancements, novel circulating biomarkers, such as soluble ST2 and GDF-15 [117], galectin-3 [118], and extracellular vesicle profiles, are being investigated for their ability to reflect subclinical RV myocardial remodeling and fibrosis.

The convergence of AI-enhanced imaging and biomarker-based precision phenotyping is expected to transform the assessment of RV function. This convergence will enable the earlier identification of maladaptive remodeling, more accurate risk stratification, and improved timing and appropriate choice of interventions in patients with valvular heart disease.

## Abbreviations

AHA/ACC, American Heart Association/American College of Cardiology; CCT, Cardiac computed tomography; CMR, Cardiac magnetic resonance imaging; EF, Ejection fraction; ESC, European Society of Cardiology; FAC, Fractional area change; FWLS, Free wall longitudinal strain; GLS, Global longitudinal strain; LV, Left ventricle/left ventricular; RA, Right atrium; RV, Right ventricle/right ventricular; RVPAC, Right ventricular to pulmonary artery coupling; S^′^, tissue Doppler-derived systolic velocity of the lateral tricuspid annulus; sPAP, systolic pulmonary arterial systolic pressure; TAPSE, Tricuspid annular plane systolic excursion; TR, Tricuspid regurgitation; TV, Tricuspid valve.
